# Association between subclinical hyperthyroidism and a *PRKAR1A* gene variant in Carney complex patients: A case report and systematic review

**DOI:** 10.3389/fendo.2022.951133

**Published:** 2022-09-23

**Authors:** Hongyang Wang, Min Mao, Dongfang Liu, Lian Duan

**Affiliations:** ^1^ Department of Endocrinology, The Second Affiliated Hospital of Chongqing Medical University, Chongqing, China; ^2^ The Infirmary, Chongqing Mechanical Senior Technician School (Chongqing Mechanical Technician College), Chongqing, China; ^3^ Department of Hematology, The First Affiliated Hospital of Chongqing Medical University, Chongqing, China; ^4^ Department of Endocrinology, The Third Affiliated Hospital of Chongqing Medical University (Jie er Hospital), Chongqing, China

**Keywords:** hyperthyroidism, *PRKAR1A* gene, Carney complex, case report, systematic review

## Abstract

**Background and Objectives:**

It is currently controversial whether subclinical hyperthyroidism is associated with *PRKAR1A* gene variants. We describe a man with subclinical hyperthyroidism and a *PRKAR1A* gene variant who was diagnosed with Carney complex (CNC), and we performed a systematic review of published studies to assess the association between *PRKAR1A* gene variants and the risk of subclinical hyperthyroidism.

**Design and Methods:**

The PubMed, EMBASE, OVID, Science Direct, and gray literature electronic databases were searched for articles published from January 2002 to May 2021 using predefined keywords and inclusion and exclusion criteria. Data on thyroid function from selected studies were extracted and analyzed.

**Results:**

We identified a CNC patient with a subclinical hyperthyroidism phenotype combined with multiple components and genetic sequenced data. In a subsequent systematic review, twenty selected studies (14 case studies and 6 series studies) enrolling 23 individuals were included in the final analysis. The patient’s thyroid function data were qualitative in 11 cases and quantitative in 12 cases. The prevalence of subclinical hyperthyroidism in the CNC patients with a *PRKAR1A* gene variant, including our patient, was markedly higher than that in the normal population (12.5% vs. 2%)

**Conclusions:**

The findings of this systematic review provide helpful evidence that *PRKAR1A* gene variants and subclinical hyperthyroidism are related and suggest that subclinical hyperthyroidism may be a neglected phenotype of *PRKAR1A* gene variants and a novel component of CNC patients.

**Systematic Review Registration:**

https://www.crd.york.ac.uk/PROSPERO, identifier CRD42021197655.

## Introduction

Subclinical hyperthyroidism, with normal thyroxine and/or triiodothyronine levels and suppressed thyroid-stimulating hormone levels, includes progression to overt hyperthyroidism, cardiovascular conditions, bone loss, fractures, and dementia (1). Carney complex (CNC) is a rare multiple endocrine and nonendocrine neoplasia syndrome, described for the first time in 1985 by J Aidan Carney as “the complex of myxomas, spotty pigmentation and endocrine overactivity” (2). According to the CNC diagnostic criteria in 2001, the syndrome can affect the thyroid gland, manifesting as thyroid carcinoma or multiple hypoechoic nodules on thyroid ultrasonography in young patients (3), but the thyroid functions of these patients are rarely reported and controversial. Stratakis CA et al. reported that all patients had normal results of physical and biochemical examinations of the thyroid gland (total and free thyroxine, triiodothyronine, and thyrotropin levels) (4); however, some researchers observed thyrotoxicosis as a clinical finding of the syndrome (5). Seventy percent of CNC cases are caused by a *PRKAR1A* variant (3, 4).

The effect of *PRKAR1A* variants on thyroid function remains unknown. An animal study found that thyroid-specific ablation of mouse *PRKAR1A* caused hyperthyroidism and follicular carcinoma (6). We also reported a laboratory result with subclinical hyperthyroidism as the first diagnosis in a CNC patient whose variant was confirmed by *PRKAR1A* gene sequencing. These results led us to speculate that *PRKAR1A* gene variants may be related to thyroid function. To date, however, thyroid function has not been implicated in CNC patients with *PRKAR1A* variants.

CNC is a rare disease. To expand the number of patients, we performed a systematic review with strict inclusion and exclusion criteria to obtain original data on thyroid function in CNC patients with a *PRKAR1A* gene variant and compared the prevalence of subclinical hyperthyroidism between CNC patients with a *PRKAR1A* gene variant and the normal population. We searched the PubMed, EMBASE, OVID, Science Direct, and gray literature databases and systematically reviewed the thyroid function of CNC patients affected by a *PRKAR1A* gene variant.

## Methods

### Study participant and registration

The patient has signed informed consent forms and consented to the publication of this case report. This systematic review was registered with PROSPERO (CRD42021197655). Details of the protocol for this systematic review can be accessed at www.crd.york.ac.uk/PROSPERO/display_record.php?RecordID=197655. It was reported based on the Joanna Briggs Institute’s approach, for a systematic review of etiology (7).

### Search strategy

Electronic databases, including the PubMed, EMBASE, OVID, Science Direct, and gray literature databases, were searched for articles published from January 2002 to May 2021. The included research was strictly human research. There were no limits regarding the language of publication. The article type was limited to case and series studies. The search strategies used are shown in the **Supplementary Material**.

### Inclusion and exclusion criteria

*Inclusion criteria:* CNC patients (as diagnosed using the diagnostic criteria) (3) with thyroid function data and a *PRKAR1A* gene variant.

*Exclusion criteria:* CNC patients with a history of thyroid diseases (such as Graves’ disease, Hashimoto’s thyroiditis, subacute thyroiditis and so on) and pituitary or thyroid surgery.

### Data extraction

The first selection was performed by filtering duplicates with EndNote ×9 and manual filtering. Eligible studies were selected according to a multistep approach (title reading, abstract reading and full-text assessment) by two researchers working independently. Disagreements between the two researchers were resolved by a third researcher. The extracted data included age, sex, thyrotropin (TSH), free thyroxine (FT4), adrenocorticotropic hormone (ACTH), *PRKAR1A* gene variant, cortisol, and other relevant components of CNC.

### Risk of bias assessment

To control the risk of bias, gray literature databases were used. Because the original data of thyroid function were extracted, the bias control tool was not involved.

### Strategy for data synthesis

A meta-analysis was not performed because there were no three or more thyroid function data of CNC patients with *PRKAR1A* variant in enrolled reports that had been extracted.

### Research outcome

The main outcome was the prevalence of subclinical hyperthyroidism in CNC patients with thyroid function data and a *PRKAR1A* gene variant.

## Case presentation

A 36-year-old Chinese male was admitted to our endocrine ward in January 2018 with a 2-month history of repeated fatigue, decreased TSH (0.01 mIU/L), and normal free triiodothyronine (FT3), FT4, and thyroid color Doppler ultrasound. He was treated with methimazole 5 mg QD. He had a history of hypertension for 2 years and was being treated with metoprolol succinate, valsartan, and amlodipine tablets. Cardiac ultrasound suggested thickening of the ventricular septum. He had moderate sleep apnea syndrome. He was married and had one child. Many people in his family had pigmentation.

**Figure 1:**
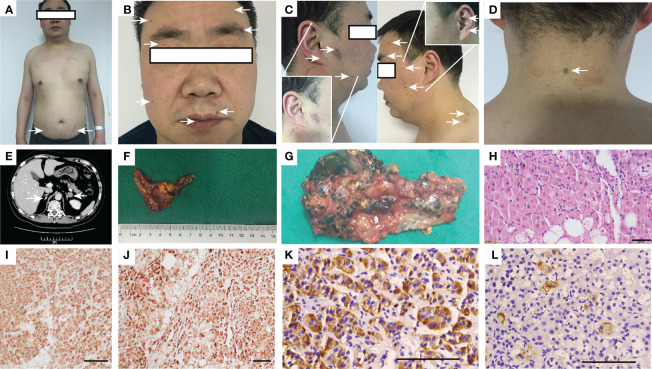
Appearance of the patient, adrenal CT, postoperative gross, HE and histochemical staining. **(A)** Appearance and lower abdomen purple streaks; **(B, C)** Lip mucosa and multiple skin spot-like pigmentation; **(D)** Posterior neck skin myxoma; **(E)** Adrenal CT: bilateral adrenal nodules, the left side diameter is 1.9 cm, the enhanced CT value is approximately 40 HU; **(F, G)** Postoperative gross after left adrenalectomy: The diseased tissue is grayish-yellow–gray–brown, 4.5•2.5•2 cm, multinodular, some cells have pigmentation, and the surrounding cortex is squeezed and thinned; **(H)** After hematoxylin-eosin staining, brown are seen particles •400; **(I–L)** Immunohistochemistry of SYN (•200), Inhibin-α (•200), CYP11B1and CYP11B2 (•400). CT, Computed tomography; HE, hematoxylin-eosin.

His heart rate was 119 times/min, his blood pressure was 122/95 mmHg, his height was 167 cm, his weight was 70.3 kg, and his body mass index was 25.2 kg/m^2^. A physical examination at the time of admission revealed bruising at the elbow skin, purple streaks on the lower abdomen (**Figure 1A**), spotty pigmentation of the lip mucosa and multiple skin areas (**Figures 1B, C**), skin myxoma of the posterior neck (**Figure 1D**), and no thyroid enlargement.

His white blood cell count was 10.15 × 10^9/L, his potassium was 3.09 mmol/l, his oral glucose tolerance test was 6.42 mmol/l at 0 h and 9.3 mmol/l at 2 h, and his growth hormone was 0.067 ng/ml. The patient’s TSH was repeatedly lower than the lower limit of the normal reference value when he was treated at other hospitals in the previous 2 months and before he was admitted to our hospital, and the fluctuation was between 0.01 and 0.02 IU/ml (**Table 1**). Preoperative assessments of the adrenal gland and thyroid function are shown in **Table 1**.

**Table 1:** Preoperative assessment and postoperative follow-up of the patient.

	Cortisol			ACTH(7-63.3 pg/ml)	UFC(3.5-45 µg/24 h)	Potassium (3.5-5.5 mmol/l)	Sodium (135-145 mmol/l)	FT3(3.8-6.0 pmol/l)	FT4(7.86-14.41 pmol/l)	TSH(0.34-5.6 mIU/l)	TRAb(0-1.75 IU/l)	TPOAb (0-9 IU/ml)	TGAb (0-4 IU/ml)
	8 am (6.71-22.54 ug/dl)	4 pm (<10.0 ug/dl)	0 am(<3.62 ug/dl)									
Baseline	25.37	25.51	23.55	6.2	316.8	3.09	140.6	5.3	12.36	0.02	<0.3	0.2	0.2
1 mg DST	22.65												
8 mg DST	24.1												
1 week after operation	16.45	15.35	15.84	3.8		3.82	141.5	4.77	10.96	0.5	/	0.2	0.3
2 months after operation	6.01	6.5	6.73	7.2	19	4.36	141.1	5.86	9.58	0.29	/	/	/
30 months after operation	472.76(177-579 nmol/l)	380.85(66-353 nmol/l)	518.08(<100 nmol/l)	2.52(5-60 pg/ml)	/	/	/	5.06(3.1-6.8 pmol/l)	15.48(12-22 pmol/l)	0.364(0.27-4.2 mIU/l)	0.94(0-1.75 IU/l)	8.76 (0-40 IU/ml)	7.97 (0-70 IU/ml)

ACTH, adrenocorticotropic hormone; DST, dexamethasone suppression test; FT3, free triiodothyronine; FT4, free thyroxine; TSH, thyrotropin; TRAb, thyrotropin receptor antibody; UFC, urinary free cortisol.

Because cortisol is not suppressed by low-dose dexamethasone and low levels of ACTH, the patient was diagnosed with ACTH-independent Cushing syndrome. Computed tomography (CT) of the adrenal glands showed nodules on the left lateral adrenal branch and nodular thickening of the medial adrenal branch on the right. Enhanced scanning showed uniform enhancement (**Figure 1E**). The thyroid gland had a full shape, a smooth capsule, and a homogeneous glandular echo; color Doppler flow imaging (CDFI); the blood flow signal within the thyroid gland did not show an abnormal increase or decrease, and there was no significant difference in bilateral blood flow signals. No echogenic lesions were found in the glands. A thyroid scan was not performed because no thyroid nodules were found on thyroid ultrasound.

Pituitary magnetic resonance imaging, cardiac, breast and testicular ultrasound and bone density assessments showed no abnormalities.

**Figure 2:**
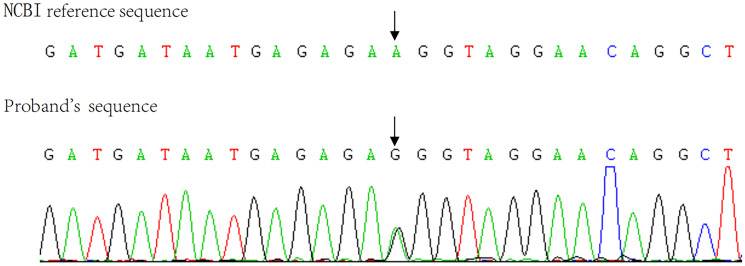
Sanger sequencing showed the patient’s C.439 A>G mutation in exon 4 of the PRKAR1A gene.

The pathological diagnosis was primary pigmented nodular adrenocortical disease (PPNAD) after left adrenalectomy (**Figures 1F–L**), postoperative gross, hematoxylin-eosin (HE), and histochemical staining). After obtaining the patient’s informed consent, direct DNA sequencing of *PRKAR1A* was performed. Sequence analysis revealed a reported heterozygous point variant at codon 439 of exon 4 (c.439 A>G) of the *PRKAR1A* gene (**Figure 2**). A diagnosis of CNC was finally made. The patient refused right adrenalectomy at the follow-up 2 months after surgery and evaluation of family members due to privacy concerns. The postoperative follow-up of the patient is shown in **Table 1**.

## Results of the systematic review

### Study identification

**Figure 3:**
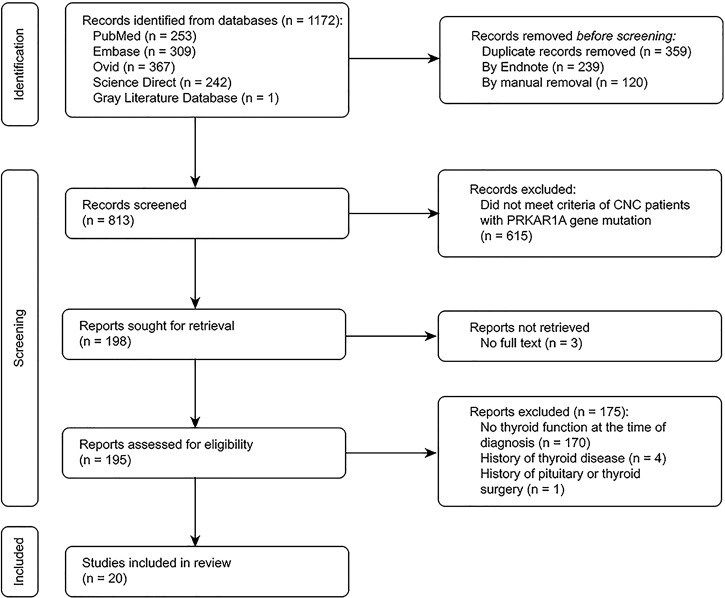
Flow chart for the systematic review.

We identified 195 potentially eligible studies on patients with a *PRKAR1A* gene variant diagnosed with CNC. Twenty studies including patients with thyroid function test results were deemed eligible. **Figure 3** describes the study selection process.

### Study characteristics

A total of 20 selected studies (14 case studies and 6 series studies) enrolling 23 individuals were included for final analysis (**Table 2**). The age of the patients ranged from infant age to 55 years. The ratio of male to female patients was 13:10. The rate of the merging PPNAD phenotype was 47.83% (11/23). At least 20 different *PRKAR1A* variants were described in the systematic review, covering six coding exons (exons 2-7), at least two intronic sequences (introns 2 and 7) and the initiator sequence (8–27).

**Table 2:** Characteristics of the included studies.

Study	Age, y/Sex	TSH	FT4	ACTH	Cortisol	*PRKAR1A* gene Mutation	CNC and other relevant components
Bilkhu et al. (2021) (8)	Infant/M	N	N	NR	N	c.549+1G>A	Dark brown macules, blue naevi, wart like appendage, testis uncertain calcification
Ralser et al. (2020) (9)	31/F	0.87 (0.27-4.2uU/ml)	NR	<1.5 (7.2-63.3 pg/ml)	36.1 (5-25 ug/dl)	a large deletion	Lentigines, most likely PMS
Chatzikonstantinou et al. (2020) (10)	53/F	3.443 (0.4-4uIU/ml)	1.3 (0.7-1.55 ng/dl)	30 (0-46 pg/ml)	15 (4.2-38.4 ug/dl)	c.431dupA	Pigmented spots, thyroid hypoechoic nodules, macules and papules, lentigines, blue nevi, cutaneous myxomas, nipple myxoma, cardiac myxomas, osteochondromyxoma or PMS
Shams et al. (2020) (11)	20/F	N	N	<5 (6-76 pg/ml)	NR	c.531_534delTGAT	Cardiac myxomas, spotty pigmentations, melanocytic nevus, Café au laitlentigo, cutaneous myxoma, thyroid nodules
Navarro et al. (2018) (12)	16/F	N	N	2 (5–49 pg/ml)	419 (220–520 nM)	c.709-7_709-2del6	PPNAD, thyroid cystic nodules
Kiriakopoulos et al. (2018) (13)	35/M	N	N	1 pg/ml	24.13-28.49-30.4 μg/dl (8:00-16:00-22:00)	c.487_488 delAC	Pigmented nevi, PPNAD
Wang et al. (2018) (14)	45/M	E	E	NR	NR	c.491_492delTG	Cardiac myxomas, pigmentation, thyroid nodules
* Cai et al. (2017) (15)	15/F	0.109 µU/ml	N	1.18-3.67-1.01 pmol/L (8:00-16:00-24:00)	29.63-26.26-36.82 µg/dl (8:00-16:00-24:00)	a 88 A to G mutation, which changes the initiator ATG to a GTG codon	PPNAD, pituitary microadenoma
Papanastasiou et al. (2016) (16)	53/M	1.6 (0.35-4.94 uIU/ml)	14.8 (9.01-21pmol/l)	5.5 (9-52 pg/ml)	389 (8AM:138-690 nmol/l)	c.172G>T	Cardiac myxomas, intestinal polyp, spotty pigmentation(lentigines), cutaneous myxomas, thyroid nodules, microcalcifications of the testes, PPNAD
Mineo et al. (2016) (17)	40/M	0.67 μIU/ml	0.8 ng/dl	≤2.0 pg/ml	24.1μg/dl	c.49G>T	Colon cancer, spotty pigmentation, PPNAD
Sun et al. (2015) (18)	20/M	E	E	NR	NR	heterozygous mutation	Sertoli cell tumor of testis, cardiac myxoma, pigmentation, cutaneous myxomas, thyroid nodules
Sun et al. (2015) (18)	48/F	N	N	N	NR	heterozygous mutation	Cardiac myxomas, cutaneous myxomas, pigmentation, thyroid nodules
Jang et al. (2015) (19)	22/M	N	N	2.6 pg/ml	29.37 mg/dl	c.441-2A>G	PMS, PPNAD, LCCSCT, myxoid liposarcoma, skin myxomas, skin pigmentations
Álvaro J et al. (2013) (20)	55/F	2.99 (0.4-4 uIU/ml)	0.759 (0.8-1.9 ng/dl)	21.3 (10-46 ug/l)	12.9 (5-25 ug/dl)	a mutation	Cardiac myxomas, acromegaly, pituitary macroadenoma, breast carcinoma, skin myxomas, nevi, lentiginosis, breast fibroadenomas, colon adenomatous polyps, colon adenocarcinoma, thyroid nodules
Briassoulis et al. (2012) (21)	13/F	2.09 (0.4-4 mIU/ml)	NR	NR	3.5 (8AM:5-25 ug/dl)	c.418_419delCA	Hyperpigmented skin spots, cardiac myxomas
Briassoulis et al. (2012) (21)	29/F	2.29 (0.4-4 mIU/ml)	NR	16.3 (8AM:9-52 pg/ml)	260 (8AM:5-25 ug/dl)	c.491_492delTG	Cranial nerve schwannoma, cardiac myxoma, PPNAD, freckles/lentigines, breast fibroadenomas, thyroid nodule
Briassoulis et al. (2012) (21)	32/F	1.05 (0.4-4 mIU/ml)	NR	10.2 (8AM:9-52 pg/ml)	9.9 (8AM:5-25 ug/dl)	c.177+1G>A	Cardiac myxomas, PPNAD, freckles/lentigines, possible pituitary adenoma
Peck et al. (2010) (22)	17/F	0.97 (0.4-4 mIU/l)	1.0 (0.6-1.6 ng/dl)	<5 (8AM:10-60 pg/ml)	16.9 µg/dl	c.177+3 A>G	Brown-black nevi, PPNAD
Courcoutsakis et al. (2009) (23)	12/M	N	N	NR	NR	c.682C>T	LCCSCT, thyroidal nodule
Vandersteen et al. (2009) (24)	15/M	N	NR	N	N	R288X mutation	Cardiac myxomas, angiomyxoma, lentigines, testicle calcified Sertoli tumour
* Sasaki et al. (2008) (25)	27/F	0.28 (0.35–4.94 µU/ml)	11.2 (9.0–19.0 pmol/L)	<1.1pmol/L	562.8-631.8-656.6 nmol/L (8:00-14:00-23:00)	c.597delC	Pigmented spot, PPNAD, GH-producing pituitary adenoma
Urban et al. (2007) (26)	9/M	N	N	Low normal or subnormal ACTH levels (7.0–16.0 pg/ml)	Markedly elevated at all time points (216–318 ng/ml)	R96X CGA→TGA	Spotty pigmentation, blue nevi, eyelid myxoma, pituitary macro-adenoma, thyroid nodule, PPNAD
Carrasco et al. (2006) (27)	34/F	2.5 (0.4-4.5 μUI/ml)	1.0 (0.9-1.5 ng/dl)	<10 (15-37 ug/ml)	9.2 (7-22 ug/dl)	578 to 579del TG mutation	Spotty pigmentation, cardiac myxoma, thyroid nodules, mammary fibroadenomas, trigeminal schwannoma, subclinical acromegaly

*Subclinical hyperthyroidism patients.

CNC, Carney Complex; E, Euthyroid; F, Female; GH: Growth Hormone; LCCSCT, Large Cell Calcifying Sertoli Cell Tumor; M, Male; N, Normal; NR, Not Reported; PPNAD, Primary Pigmented Nodular Adrenocortical Disease; PMS, Psammomatous Melanotic Schwannoma.

### Thyroid function

The patient’s thyroid function data were qualitative in 11 cases and quantitative in 12 cases. Among the patients, two (highlighted with * in **Table 2**) had subclinical hyperthyroidism (15, 25), and 21 had normal thyroid function (**Table 2**). With the addition of our patient, the prevalence of subclinical hyperthyroidism was 12.5% (3/24) in the CNC patients with thyroid function results and a confirmed *PRKAR1A* gene variant.

## Discussion

We reviewed the thyroid hormone levels of CNC patients with thyroid function affected by *PRKAR1A* gene variants. The prevalence of subclinical hyperthyroidism in this populations is much higher than that in the general population, which is approximately 1% to 2% (28). This finding suggests that *PRKAR1A* gene variants may be related to subclinical hyperthyroidism, and this phenotype may be a common phenotype that is overlooked in CNC patients.

CNC is an autosomal dominant inherited multiple tumor syndrome with symptoms and signs including spotty skin pigmentation, heart and skin myxoma, endocrine overactivity, psammomatous melanotic schwannoma (PMS), etc. Spotty skin pigmentation is the most common clinical manifestation, appearing in approximately 77% of cases, and thyroid nodules or cancer occurs in 5% of cases (3). However, the thyroid function of these patients is rarely reported and remains controversial. We believe that *PRKAR1A* variant may be associated with abnormal thyroid function. First, subclinical hyperthyroidism was the first symptom in our patient associated with a *PRKAR1A* gene variant. Second, research has shown that mice with an ablated *PRKAR1A* gene and lower TSH levels have not only thyroid follicular carcinoma but also hyperthyroidism (6). Third, when J Aidan Carney summarized the spectrum of thyroid gland pathology in CNC patients, he also thought that thyrotoxicosis should be added to the clinical findings associated with the syndrome (5). However, he did not explore whether subclinical hyperthyroidism is related to *PRKAR1A* gene variants. These findings inspired us to explore the relationship between subclinical hyperthyroidism and the *PRKAR1A* gene. Because CNC patients are rare, a systematic review with strict inclusion and exclusion criteria was performed to gather all published studies worldwide to analyze the thyroid function of CNC patients affected by *PRKAR1A* gene variants. Considering that the confirmed diagnostic criteria for this syndrome were published in 2001, we excluded studies before 2001. To reduce bias and confounding factors, we excluded patients who had been diagnosed with thyroid disease and had affected thyroid function due to pituitary and thyroid surgery, as well as those with other CNC gene variants, such as *PRKACA* encoding PKA catalytic subunit α, *PRKACB* encoding PKA catalytic subunit β, *PDE11A* encoding phosphodiesterase expressed in the adrenal cortex and *PDE8B* encoding another phosphodiesterase (2), which may affect thyroid function.

Our systematic review is helpful for understanding the rare causes of hyperthyroidism. The main causes of hyperthyroidism are Graves’ disease, subacute thyroiditis, Hashimoto’s thyroiditis, excessive iodine intake, TSH tumor, thyroid hormone resistance, painless thyroiditis, thyroid follicular cancer metastasis invasion, etc. (29). The causes of subclinical hyperthyroidism are the same as the causes of overt hyperthyroidism (1). Even though we know that patients with CNC caused by *PRKAR1A* gene variants can have involvement of the thyroid gland, the rate of thyroid function testing in this population is low because it is not widely known that *PRKAR1A* gene variants may affect thyroid function. We describe a case of a Chinese man with a *PRKAR1A* gene variant and normal thyrotropin receptor antibody (TRAb), whose diagnosis of CNC was delayed, with a final diagnosis 2 months after the initial misdiagnosis and treatment for Graves’ disease. Because the patient had no typical symptoms of thyrotoxicosis and because of the lack of understanding that *PRKAR1A* gene variants may lead to subclinical hyperthyroidism, decreased TSH is easily considered by clinicians as method-specific interference and overlooked. This makes the diagnosis and treatment complicated, leading to the delayed diagnosis of CNC. The prevalence of subclinical hyperthyroidism in the specific population pooled in this systematic review was much higher than that in the general population, suggesting that *PRKAR1A* gene variants may be the cause of subclinical hyperthyroidism. Three patients in our systematic review all manifested decreased TSH but no thyroid nodules. Similar to Stergiopoulos et al., the biochemical indicators GH and IGF-1 were elevated, but no adenomas were found on imaging, which may be because hyperplasia most likely precedes the formation of GH-producing adenomas in CNC patients (30). However, we found that 11 patients in our systematic review with thyroid nodules did not manifest decreased TSH. The change in growth hormone axis status is not completely similar to subclinical hyperthyroidism in CNC patients, suggesting that their pathogenic mechanism needs further study.

The PKA holoenzyme is a heterotetramer composed of two regulatory subunits, each of which is bound to one catalytic subunit (31). Indeed, despite the existence of four regulatory subunits of PKA (*PRKAR1A*, *PRKAR1B*, *PRKAR2A*, and *PRKAR2B*), both cloning and gene knockout studies have demonstrated that *PRKAR1A* is critical for maintaining the PKA response to cAMP by regulating free catalytic subunits, especially in adrenocortical cells (32). More than 130 different variants of the *PRKAR1A* gene have been described to date in over 400 families of different ethnic origins with CNC (2). The CNC gene located at 17q22-24 was identified in 2000 as the tumor suppressor gene *PRKAR1A* encoding the regulatory subunit type 1α of protein kinase A (33, 34). When gene variants result in abnormal protein synthesis of regulatory subunit type 1α, they can lead to altered activity of protein kinase A, with consequently increased cell proliferation and tumorigenesis (35). The variant site in our case was in exon 4, and the variant sites in the other two subclinical hyperthyroidism patients were located in the promoter and exon 6. The variant sites and other components of CNC vary, suggesting that there may be no hot spot variant. How *PRKAR1A* gene variants cause subclinical hyperthyroidism remains unclear. Elevated levels of TSH stimulating PKA activity *via* the activation of adenylyl cyclase and the production of cAMP are associated with the development of thyroid cancer in humans (36). However, a mouse model of elevated TSH signaling in a genetic wild-type background did not develop thyroid cancer (37). Pringle, D. R. et al. observed that thyroid-specific ablation of *PRKAR1A*, exhibiting low levels of TSH, leads to hyperthyroidism and thyroid cancer and speculated that TSH may be suppressed by activating alternative pathways along with activated PKA that provide negative feedback on cell growth. This hypothesis may explain why mice with PKA activation develop FTC, while tumors driven by elevated TSH do not develop cancers (6). Whether the mechanism of *PRKAR1A* variant-induced low TSH levels in CNC is the same as the *PRKAR1A* tumorigenic mechanism needs further investigation.

Subclinical hyperthyroidism may be influenced by not only excessive glucocorticoid suppression but also *PRKAR1A* gene variants. It is well known that glucocorticoids at high levels, such as those in Cushing’s syndrome, induce the suppression of TSH secretion (38). Bilateral adrenalectomy used to be considered the treatment of choice for patients with overt Cushing’s syndrome (39). The suppression of TSH is dissolved along with decreases in the high cortisol level after adrenalectomy. Although there is no mention of *PRKAR1A* gene variants, ACTH-independent Cushing’s syndrome results in postoperative adrenal insufficiency with inappropriate secretion of TSH (high TSH) after complete adrenalectomy (40). Similar to this study (15), our patient after unilateral adrenalectomy showed decreases in high cortisol levels and increased ACTH, but TSH of our patient was still below the lower limit of normal reference values, suggesting that preoperative subclinical hyperthyroidism may be mainly caused by *PRKAR1A* gene variants, not by excessive glucocorticoid suppression. Unfortunately, no study has observed changes in TSH after bilateral adrenalectomy in subclinical hyperthyroidism CNC patients caused by *PRKAR1A* gene variants. This warrants confirmation that the suppression of TSH is mainly caused by variants of the *PRKAR1A* gene in a follow-up prospective observation.

Hyperthyroidism is a common endocrine dysfunction. The prevalence of hyperthyroidism is 0.8% in Europe and 1.3% in the USA general population (41). This study found that in CNC patients, *PRKAR1A* gene variants led to a significantly higher prevalence of subclinical hyperthyroidism than that in the general population. Even so, subclinical hyperthyroidism in this specific group may still be underestimated. 1. We found that many patients who may suffer from hyperthyroidism were excluded for different reasons, such as a history of pituitary or thyroid surgery and thyroid diseases. For example, Hernandez-Ramirez LC et al. (42) reported that a patient was diagnosed with central hypothyroidism and corticotropinoma before the subclinical hyperthyroidism diagnosis, and this patient was excluded due to a history of pituitary surgery. In another study (5), the diagnosis was thyrotoxicosis; however, Graves’ disease is mentioned in the following table, which makes us suspect that thyrotoxicosis was caused by Graves’ disease. Due to the lack of specific key thyroid function data, such as TSH and TRAb, to identify the cause of thyrotoxicosis, we excluded this patient after discussion. Piper, S. N. et al. (43) believed that transient hyperthyroidism may have occurred in a patient with CNC. We excluded this patient because the study was conducted before 2001. The exclusion of patients who may have had subclinical hyperthyroidism led to fewer thyroid function data available for the final analysis. 2. Autopsy research reports show that most CNC patients have pathological changes in the adrenal glands and other organs, indicating that many patients with abnormal findings that can only be diagnosed by endocrinological tests have been overlooked (25). Other low-incidence phenotypes of CNC may be temporarily asymptomatic (subclinical hyperthyroidism often has no typical hypermetabolic symptoms of overt hyperthyroidism) orinaccurate assessment of endocrine parameters (25), and many components can be tested only by endocrinology (such as thyroid function abnormalities that could be diagnosed through testing for thyroid function), causing these patients to be ignored. 3. Due to incomplete penetrance at the time of diagnosis and continued follow-up, subclinical hyperthyroidism may gradually manifest. For example, J Aidan Carney et al. (5) believed that thyroid disorder was a late-developing feature of CNC, and that abnormal thyroid function may appear after CNC diagnosis. Similar to this study (44), the accurate prevalence of subclinical hyperthyroidism in CNC patients with *PRKAR1A* in the real world should be investigated in a multicenter prospective long-term follow-up study.

Our study has some limitations that lead to a deviation from the real-world population prevalence of subclinical hyperthyroidism. 1. Due to the retrospective nature of the study, insufficient thyroid function data reduced the robustness of the results. 2. Even if thyroid function is checked, there are still qualitative data described by words such as “normal” and “euthyroid”. We did not contact the researchers to ask for patients’ concrete TSH and FT4 levels, so they could not be included in the meta-analysis, which weakened the performance of the results. 3. Studies for which the full text was not available were not included in the analysis, and there may be a bias that affects the performance of the results. Further prospective studies with a large number of patients are needed to confirm our findings.

In conclusion, the results of our systematic review showed that the prevalence of subclinical hyperthyroidism in CNC patients with *PRKAR1A* gene variants is higher than that in the normal population. To the best of our knowledge, this is the first discovery that *PRKAR1A* gene variants may be related to subclinical hyperthyroidism, and our results also suggest that subclinical hyperthyroidism may be a new component of CNC patients with a *PRKAR1A* gene variant; however, confirmatory prospective studies are needed.

## Data availability statement

The datasets presented in this study can be found in online repositories. The names of the repository/repositories and accession number(s) can be found in the article/**Supplementary Material**.

## Author contributions

LD conceptualized the research question and study design. HW informed the search strategy. MM informed the analytic plan and drafted and edited the manuscript. LD and DL revised the manuscript. All authors listed have made a substantial, direct, and intellectual contribution to the work and approved it for publication.

## Funding

This work was supported by grants from Chongqing medical scientific research project (Joint project of Chongqing Health Commission and Science and Technology Bureau) (2022GDRC016), Key Laboratory Incubation Project of the Third Affiliated Hospital of Chongqing Medical University (KY19025), and High-level Medical Reserved Personnel Training Project of Chongqing (CQSZQNYXGDRC201829).

## Conflict of interest

The authors declare that the research was conducted in the absence of any commercial or financial relationships that could be construed as a potential conflict of interest.

## Publisher’s note

All claims expressed in this article are solely those of the authors and do not necessarily represent those of their affiliated organizations, or those of the publisher, the editors and the reviewers. Any product that may be evaluated in this article, or claim that may be made by its manufacturer, is not guaranteed or endorsed by the publisher.
